# Effects of water drinking test on ocular blood flow waveform parameters: A laser speckle flowgraphy study

**DOI:** 10.1371/journal.pone.0181512

**Published:** 2017-07-24

**Authors:** Mehwish Saba Bhatti, Tong Boon Tang, Augustinus Laude

**Affiliations:** 1 Centre for Intelligent Signal and Imaging Research (CISIR), Universiti Teknologi PETRONAS, Bandar Seri Iskandar, Perak Darul Ridzuan, Malaysia; 2 National Healthcare Group Eye Institute, Tan Tock Seng Hospital, Singapore, Singapore; Bascom Palmer Eye Institute, UNITED STATES

## Abstract

The water-drinking test (WDT) is a provocative test used in glaucoma research to assess the effects of elevated intraocular pressure (IOP). Defective autoregulation due to changes in perfusion pressure may play a role in the pathophysiology of several ocular diseases. This study aims to examine the effects of WDT on ocular blood flow (in the form of pulse waveform parameters obtained using laser speckle flowgraphy) to gain insight into the physiology of ocular blood flow and its autoregulation in healthy individuals. Changes in pulse waveform parameters of mean blur rate (MBR) in the entire optic nerve head (ONH), the vasculature of the ONH, the tissue area of the ONH, and the avascular tissue area located outside of the ONH were monitored over time. Significant increases in the falling rate of MBR over the entire ONH and its tissue area and decreases in blowout time (BOT) of the tissue area were observed only at 10 minutes after water intake. Significant increases in the skew of the waveform and the falling rate were observed in the vasculature of the ONH at 40 and 50 minutes after water intake, respectively. In the avascular region of the choroid, the average MBR increased significantly up to 30 minutes after water intake. Furthermore, the rising rate in this region increased significantly at 20 and 40 minutes, and the falling rate and acceleration-time index were both significantly increased at 40 minutes after water intake. Our results indicate the presence of effective autoregulation of blood flow at the ONH after WDT. However, in the choroidal region, outside of the ONH, effective autoregulation was not observed until 30 minutes after water intake in healthy study participants. These pulse waveform parameters could potentially be used in the diagnosis and/or monitoring of patients with glaucoma.

## Introduction

Age-related macular degeneration, glaucoma, and diabetic retinopathy are major sight threatening diseases which are linked to ocular perfusion irregularities [1–3]. Approximately 196 million people between the ages of 30 and 97 years will be affected by age-related macular degeneration by 2020 [4]. The prevalence of glaucoma in people aged between 40 and 80 years is estimated to be over 64.3 million worldwide [5]. There are approximately 93 million people (aged 20 to 79 years) affected with diabetic retinopathy worldwide [6]. Other risk factors of ocular perfusion irregularities include: intraocular pressure (IOP); blood pressure, which leads to changes in perfusion pressure and blood flow; aging; and sometimes, reduced cerebrospinal fluid pressure [7–15]. Most medical and surgical approaches aim to prevent the development of, or delay the progression of glaucomatous optic neuropathy by lowering IOP [16,17]. However, despite achieving a reduction in IOP, optic nerve damage may persist in some patients and eventually result in progressive vision loss [18–20]. In recent studies, the hypothesis that glaucoma could be attributed to changes in blood flow to the optic nerve, vascular dysregulation and autoregulatory capacity has been suggested [21–24]. Most of the literature in this area focuses on hemodynamic parameters relating to homeostasis. However, assessment of changes in blood flow in response to standardized stimuli may also lead to a better understanding of optic neuropathy [25]. Healthy eyes with no ocular pathologies are able to maintain a constant blood flow despite fluctuations in ocular perfusion pressure [26,27]. However, a defective autoregulatory response to changes in ocular perfusion pressure has been observed in a selected group of patients with open-angle glaucoma [28]. Perturbation in this autoregulatory mechanism could, therefore, explain the occurrence of ocular pathologies, their progression, and the inability of a reduction in IOP to prevent or delay neurodegeneration.

The water-drinking test (WDT), which alters IOP and ocular perfusion pressure, is a simple provocative test that enables the assessment of autoregulatory function in both non-glaucomatous eyes and in those with ocular pathologies such as glaucoma. As originally proposed in the 1920s by Marx [29], WDT enables the detection of impairments in ocular fluid dynamics within minutes by observing the ocular response to consumption of a liter of water [30]. Fluctuations in IOP and/or ocular perfusion pressure, owing to the inability of eyes to withstand this form of stress, might reflect the presence of a specific condition such as glaucoma [31,32]. In addition, WDT has also been used to assess the outcomes of therapeutic interventions among patients with glaucoma [33–35]. Some investigators have attempted to explain the changes in ocular fluid dynamics observed during WDT through mechanisms such as disturbance of the blood-ocular osmotic pressure gradient, autonomic nervous stimulation, elevated episcleral venous pressure, changes in aqueous outflow and an increase in choroidal thickness [31,33,36–38]. Other effects, such as an increase in corneal compensated IOP during the first 10 minutes following water loading have also been proposed [39]. However, the physiological changes that take place in the eye during WDT are still not fully understood. In this research, we attempted to understand the physiology of WDT in the context of ocular blood flow in healthy human eyes by monitoring ocular blood flow parameters obtained using laser speckle flowgraphy (LSFG). Data on these parameters of ocular blood flow were obtained at two regions of interest: the optic nerve head (ONH) and the tissue area in the choroid away from the ONH. The ONH was selected as a region of interest because it is the principal site of glaucomatous neurodegenerative changes [2,40]. The tissue area in the choroidal region was selected due to differences in various cellular properties and in the vascular diameter relative to the ONH [41,42]. We hypothesize that the ocular blood flow pulse waveform parameters can act as useful predictive biomarkers for the assessment of alterations in ocular physiology attributed to changes in perfusion or IOP.

## Materials and methods

### Data collection

The experimental protocols used in this study abide by the tenets of the Declaration of Helsinki. The approval of this study and the procedure of getting consent from participants were obtained from the ethics committee of the Universiti Teknologi PETRONAS, Malaysia. All study participants provided written informed consent to participate in this study. Investigations were conducted on 16 paid participants (10 women and 6 men) with no known history of any ophthalmological disorders, autonomic dysfunction, or cardiovascular disease. The mean age of participants in this study was 29 ± 7 years (range: 22 to 36 years). Moreover, all participants were non-smokers, and were not taking any medication at the time of the study or for at least 3 weeks prior to enrollment. All participants were advised to refrain from any food, alcohol or caffeine consumption, or exercise for at least 2 hours prior to undergoing the experiment. The experimental objectives, techniques and risks were explained to each participant prior to enrollment.

### Laser speckle flowgraphy

**Fig 1:**
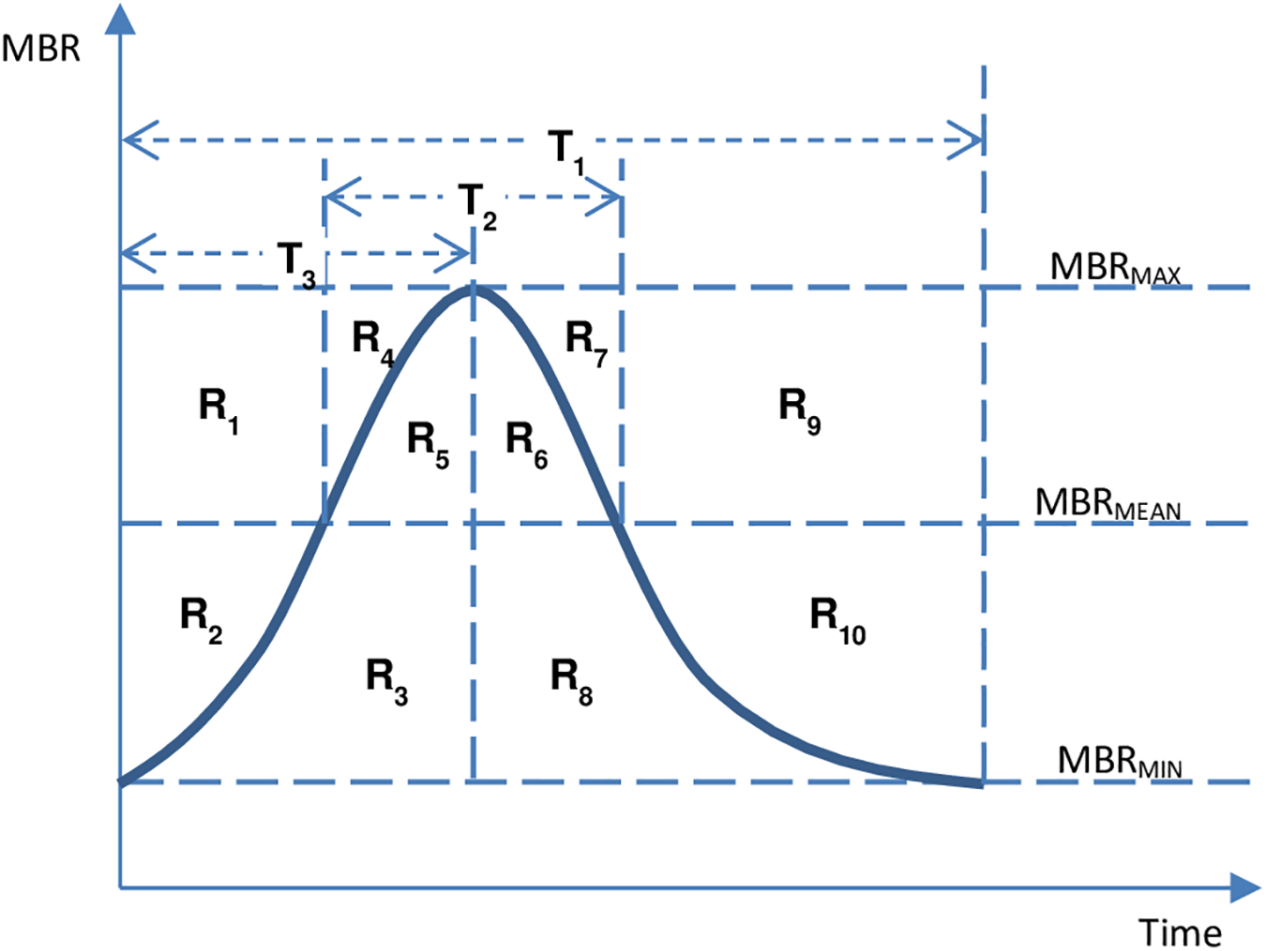
Spatiotemporal divisions of a typical mean blur rate (MBR) waveform. Regions R_1_-R_10_ indicate the various regions above and below the average pulse waveform, and T_1_-T_3_ indicate distinct time intervals. T_1_ refers to the time taken to complete one heartbeat, T_2_ is the time interval where the value of an average pulse wave parameter is greater than the average MBR, and T_3_ is the time taken to reach the maximum MBR value [43].

Laser speckle flowgraphy (LSFG) is a non-invasive modality that enables measurement of ocular blood flow. The LSFG system uses a regular charge-coupled-device camera to capture a frame size of 750 x 360 pixels at a temporal frame rate of 30 frames per second. The accompanying software calculates the mean blur rate (MBR) as an indicator of relative blood flow volume. After manually defining the optic nerve head (ONH) and the avascular region as the two regions of interest, LSFG analysis software (Softcare Co., Ltd., Fukuoka, Japan) can be used to compute an MBR for the entire ONH region, ONH blood vessels, ONH tissue and the avascular region. Consequently, cardiac cycles are computed and MBR pulse waveform parameters are analyzed: skew, blowout time (BOT), rising rate, falling rate, and acceleration-time index (ATI). Further explanation of the pulse waveform parameters is provided below, as indicated in the regions and time-slices labeled in Fig 1 [43]. The figure (Fig 1) shows a schematic waveform graph of average MBR parameter against time. The maximum, minimum, and mean values of MBR are reported as MBR_MAX_, MBR_MIN_, and MBR_MEAN_, respectively. The time to achieve one complete cycle is designated as T_1_, the time to pass the MBR_MEAN_ while rising and falling as T_2_, and the time to reach MBR_MAX_ as T_3_. The rest of the waveform is divided into ten distinct regions (R_1_ to R_10_).

#### Skew

Skew is calculated from the average pulse waveform shape, and equals 0 if perfectly symmetrical. However, skew is positive if the waveform at the peak is tilted towards the left and negative if the peak is tilted to the right.

#### BOT

This value indicates how long blood flow is maintained at a value higher than the mean value of blood flow during each heartbeat. BOT is computed as:

$$BOT=100\times T_{2}÷T_{1}$$ (1)

#### Falling rate

This value indicates the rate of decrease in blood flow with each heartbeat. Falling rate is computed as:

$$Falling\;rate=25\times (R_{6}+R_{8})÷(R_{6}+R_{7}+R_{8}+R_{9}+R_{10})$$ (2)

#### Rising rate

This value indicates the rate of increase in MBR beat waveform, and is computed as:

$$Rising\;rate=25\times (R_{3}+R_{5})÷(R_{1}+R_{2}+R_{3}+R_{4}+R_{5})$$ (3)

#### ATI

This value indicates the time taken to reach the peak MBR value in the mean MBR, and is computed as:

$$ATI=T_{3}÷T_{1}$$ (4)

**Fig 2:**
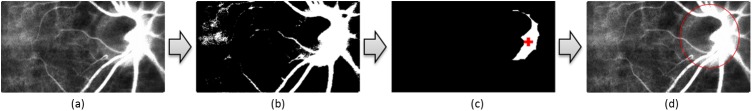
Dynamic localization of optic nerve head (ONH). (a) Original frames from the CCD camera recording were converted to grayscale. (b) A threshold was applied to generate a binary image. (c) A disk-shaped structuring element was applied after eroding the image and a centroid (‘+’) was obtained. (d) ONH position is localized relative to the location of the centroid.

The accompanying software is sometimes unable to automatically locate and track the position of the ONH for all the recorded measurements. Therefore, an adaptive localization method was used for locating the ONH. The grayscale images were obtained from the accompanying software and further converted to binary images using threshold at average intensity values from the observed intensity range. Morphological operators were applied to remove smaller objects and structures from the binary images. This is carried out by convolution using a disc-shaped structuring element. The most significant size of the structuring element inferred from the dataset is 25 pixels. Regional properties, area and centroid, are obtained from labeled objects generated after morphological operation. The centroid of the largest cluster is assumed to be the centroid of the vascular ONH, which is the largest cluster obtained, and subsequently used to identify the position of the entire ONH. The ONH position is dynamically discovered relative to the location of the centroid, which can be further adjusted to compensate for individual variations. The steps involved in dynamically locating the ONH are shown in Fig 2.

### Experiment design flow

**Fig 3:**
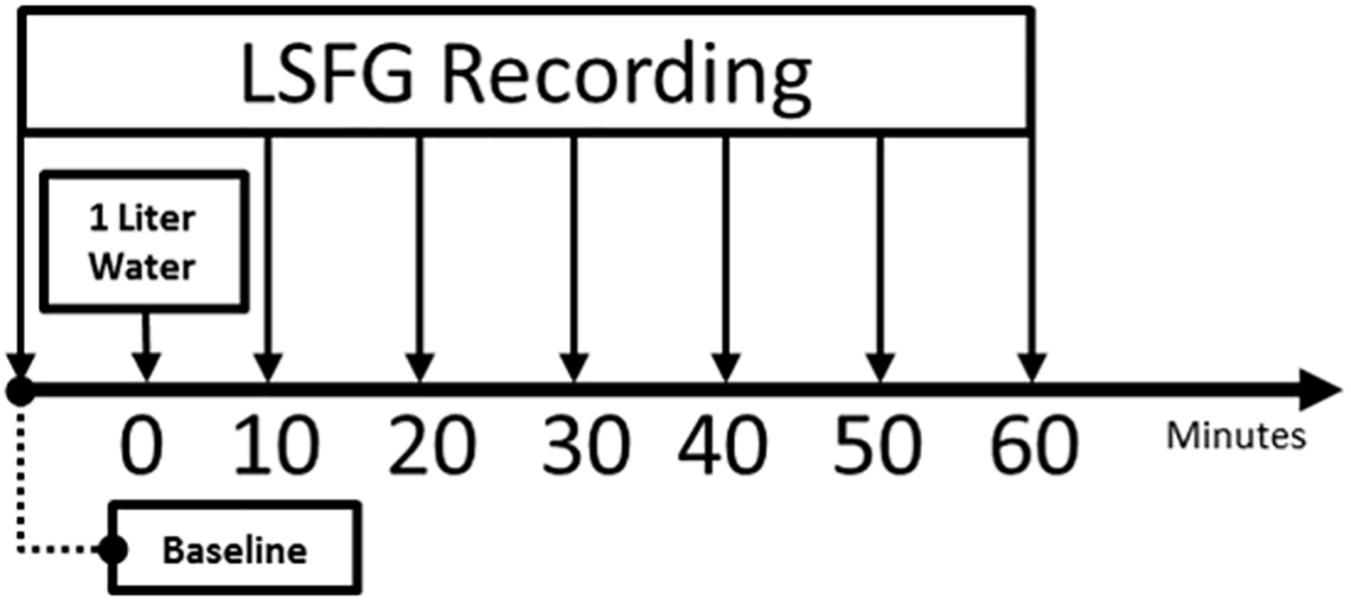
Timeline for LSFG recordings according to experiment design. A baseline LSFG recording before drinking 1 liter of water, followed by six further recordings at 10-minute intervals.

All subjects were familiarized with the LSFG-NAVI system (Softcare Co., Ltd., Japan) and the various procedural details. The LSFG-NAVI system was adjusted according to the eye position of each participant, and each was given sufficient time to relax before the experiment commenced. All the recordings were conducted in a darkroom. The first LSFG recording was performed before the participant drank 1 liter (1000 mL) of water. The participant was instructed to consume the entire liter within a few minutes. The next LSFG recording was performed 10 minutes after commencement of water intake. Five subsequent recordings were obtained at 10-minute intervals until 60 minutes after water intake. The time intervals for all recordings are shown in Fig 3. Three consecutive recordings were acquired at each time point, and the average of these three values was used for further analysis.

### Data analysis

**Fig 4:**
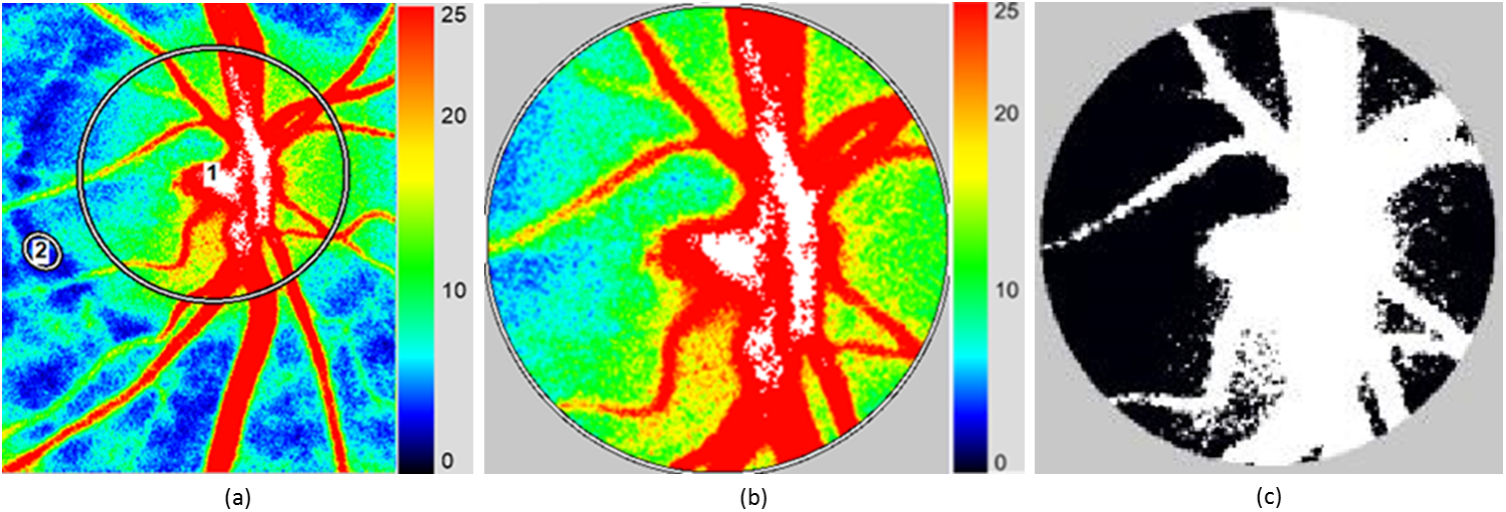
Selected regions of interest. (a) Whole ONH region (‘1’) in the heartbeat image, and the avascular region (‘2’), (b) ONH region extracted from the whole frame for cross-sectional analysis, and (c) Segmentation of the ONH into a vascular (white) and tissue area (black). The white patch in (a) and (b) refers to regions with MBR > 80.

The LSFG-NAVI system provided MBR pulse waveforms as measurements of ocular blood flow. Pulse waveform parameters were obtained using the proprietary analysis software provided with the LSFG-NAVI system [43]. MBR was computed for two regions of interest in each participant: the entire ONH and the avascular region, as shown in Fig 4(A). The ONH region, shown in Fig 4(B), was segmented into its constituent vascular and tissue areas, as shown in Fig 4(C). The pulse waveform parameters of the MBR waveform for the whole ONH, the vascular part of the ONH, the tissue part of the ONH and the avascular region were exported from the LSFG analysis software for further analysis. Moreover, heart rate was also computed from the MBR waveform using the LSFG analysis software, and is reported in this study.

The values were normalized according to baseline readings in each participant, and statistical analysis was performed to assess any significant difference. Statistical analyses were conducted using SPSS statistical software. Based on visual inspection, utilizing histograms, QQ plots and box plots, the data was found not to follow normal distribution. Therefore, a nonparametric test called Wilcoxon Signed Rank test is carried out on the dataset, and asymptotic significance (2-tailed) was computed at P < 0.05. Wilcoxon signed-rank test is as powerful for non-parametric data as is the t-test for parametric data [44].

## Results

**Fig 5:**
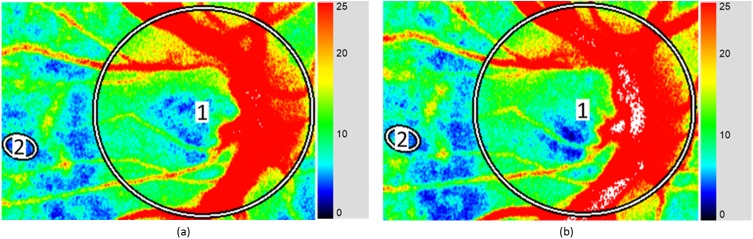
Raw data from one of the participants. (a) Baseline recording, where waveform parameters for the entire ONH were recorded (‘1’): average MBR = 20.50, skew = 11.27, BOT = 52.53, rising rate = 11.93, falling rate = 12.27, and ATI = 30.23; and from the avascular region (‘2’): average MBR = 4.17, skew = 14.50, BOT = 46.67, rising rate = 10.93, falling rate = 13.63, and ATI = 26.90 (b) Recording taken 10 minutes after water intake, where waveform parameters for the entire ONH were recorded (‘1’): average MBR = 24.57, skew = 11.77, BOT = 54.23, rising rate = 13.17, falling rate = 12.67, and ATI = 29.43; and from the avascular region (‘2’): average MBR = 6.40, skew = 11.30, BOT = 51.33, rising rate = 12.10, falling rate = 13.13, and ATI = 29.83.

Mean pulse waveform data were obtained for all participants at baseline, and a further six consecutive readings were recorded at 10-minute intervals, as shown in Fig 5. Mean values for all ocular waveform parameters analyzed in this study are summarized in Table 1. Differences for which *P* <0.05 relative to baseline values are indicated as statistically significant.

**Table 1:** Ocular pulse waveform dynamics (mean ± standard deviation) before and after the water drinking test (N = 16), where the significant differences (*P* < 0.05) are indicated.

***Region ‘1’*: *Whole ONH area***							
	**Baseline**	**10-minute**	**20-minute**	**30-minute**	**40-minute**	**50-minute**	**60-minute**
**Average**	27.02 ± 5.33	26.96 ± 5.54	27.47 ± 4.48	27.84 ± 5.35	27.12 ± 4.81	26.83 ± 4.63	27.03 ± 4.19
**Skew**	9.82 ± 1.25	10.26 ± 1.61	10.30 ± 1.04	10.11 ± 1.22	10.30 ± 1.25	10.02 ± 1.12	10.37 ± 1.31
**BOT**	57.41 ± 3.35	55.46 ± 4.31	57.29 ± 3.37	57.19 ± 3.30	56.38 ± 2.89	57.76 ± 4.02	57.03 ± 4.21
**Rising rate**	13.00 ± 1.03	13.16 ± 0.95	13.38 ± 1.17	13.32 ± 0.77	13.26 ± 1.44	13.69 ± 1.74	13.47 ± 0.89
**Falling rate**	11.63 ± 1.08	12.00 ± 1.01*	11.93 ± 0.73	11.77 ± 0.88	11.92 ± 0.84	11.97 ± 0.92	11.90 ± 0.94
**ATI**	29.94 ± 3.95	31.02 ± 2.88	29.85 ± 3.93	30.30 ± 3.28	29.76 ± 4.25	30.51 ± 5.40	29.82 ± 2.95
***Region ‘1’*: *Vascular area of ONH***							
	**Baseline**	**10-minute**	**20-minute**	**30-minute**	**40-minute**	**50-minute**	**60-minute**
**Average**	49.31 ± 11.26	48.83 ± 9.43	49.47 ± 8.68	48.52 ± 9.02	48.31 ± 8.99	48.87 ± 8.91	48.02 ± 9.34
**Skew**	9.46 ± 1.39	9.78 ± 1.74	10.05 ± 1.22	9.91 ± 1.30	10.05 ± 1.26*	9.62 ± 1.36	10.25 ± 1.45
**BOT**	58.57 ± 3.29	56.87 ± 4.45	58.09 ± 3.78	58.10 ± 3.34	57.20 ± 2.96	58.79 ± 4.12	58.19 ± 3.97
**Rising rate**	12.79 ± 1.21	12.89 ± 1.19	13.46 ± 1.28	13.30 ± 1.31	13.33 ± 1.56	13.61 ± 1.87	13.48 ± 1.32
**Falling rate**	11.46 ± 1.10	11.69 ± 1.14	11.86 ± 0.80	11.69 ± 0.91	11.83 ± 0.81	11.85 ± 1.01*	11.81 ± 0.92
**ATI**	29.62 ± 5.19	30.04 ± 3.91	29.62 ± 4.50	29.93 ± 4.74	29.69 ± 4.43	30.14 ± 5.84	29.54 ± 3.82
***Region ‘1’*: *Tissue area of ONH***							
	**Baseline**	**10-minute**	**20-minute**	**30-minute**	**40-minute**	**50-minute**	**60-minute**
**Average**	12.46 ± 2.25	12.49 ± 2.19	12.58 ± 2.27	12.49 ± 2.26	12.46 ± 2.33	12.36 ± 2.40	12.36 ± 2.48
**Skew**	10.58 ± 1.33	11.19 ± 1.58	10.95 ± 1.05	10.86 ± 1.38	10.99 ± 1.49	10.84 ± 1.45	11.07 ± 1.48
**BOT**	54.69 ± 3.64	52.12 ± 3.92*	54.72 ± 3.36	54.18 ± 3.31	53.58 ± 3.57	54.84 ± 4.55	54.48 ± 3.40
**Rising rate**	12.59 ± 0.83	12.91 ± 0.67	13.05 ± 1.00	12.91 ± 0.94	12.74 ± 1.21	13.29 ± 1.22	12.95 ± 0.57
**Falling rate**	12.02 ± 0.88	12.65 ± 0.84*	12.16 ± 0.70	12.30 ± 0.78	12.25 ± 0.95	12.25 ± 0.94	12.33 ± 0.92
**ATI**	29.76 ± 4.44	31.24 ± 3.28	29.49 ± 3.47	30.17 ± 3.01	29.25 ± 3.39	29.65 ± 4.01	29.46 ± 3.73
***Region ‘2’*: *Avascular area outside ONH***							
	**Baseline**	**10-minute**	**20-minute**	**30-minute**	**40-minute**	**50-minute**	**60-minute**
**Average**	3.45 ± 0.8961	3.97 ± 1.2016*	3.92 ± 1.0265*	3.85 ± 0.85963*	3.70 ± 1.0992	3.72 ± 1.0819	3.76 ± 1.1434
**Skew**	11.17 ± 2.3697	11.14 ± 2.6931	11.40 ± 2.3827	11.12 ± 3.1431	11.27 ± 2.3211	10.97 ± 2.4008	10.93 ± 1.6353
**BOT**	49.59 ± 6.4271	48.21 ± 6.0886	51.18 ± 7.4649	52.32 ± 7.0228	49.61 ± 5.6353	50.17 ± 5.1042	52.58 ± 6.8448
**Rising rate**	11.67 ± 1.1729	12.18 ± 1.2795	12.68 ± 1.5894*	12.23 ± 1.6897	12.59 ± 1.3525*	11.66 ± 1.5870	12.65 ± 1.7546*
**Falling rate**	12.86 ± 1.2046	13.50 ± 1.0636	13.14 ± 1.4336	13.21 ± 0.8759	13.63 ± 1.5565*	13.30 ± 1.0305	13.16 ± 1.1257
**ATI**	29.59 ± 6.1924	32.86 ± 7.0636	30.58 ± 5.6648	31.25 ± 8.2963	33.22 ± 3.8106*	30.15 ± 5.4143	32.22 ± 5.0526

*Statistically significant (*P* < 0.05)

**Fig 6:**
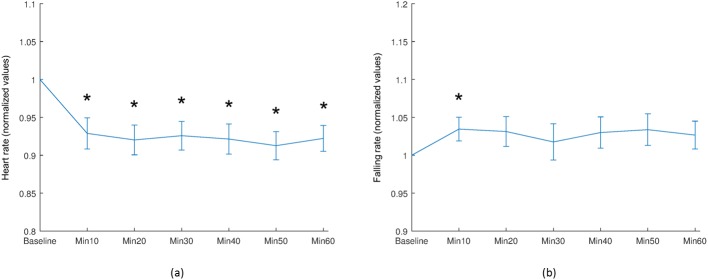
Selected mean waveform parameters of the entire ONH region (‘1’). (a) Heart rate, and (b) falling rate. Asterisk (*) indicates a statistically significant difference compared with baseline values (Wilcoxon signed-rank test: *P* <0.05) and vertical bars represent the standard errors of means.

A significant decrease in mean heart rate was observed at all time points, relative to baseline values (*P* <0.01) as shown in Fig 6(A). A statistically significant increase in falling rate was observed at a 10-minute interval after water intake (*P* = 0.039), as shown in Fig 6(B).

**Fig 7:**
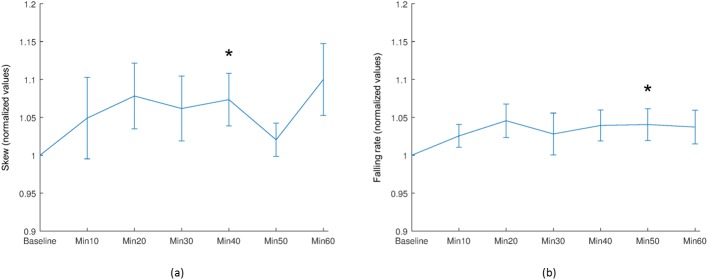
Selected mean waveform parameters of the vascular ONH. (a) Skew, and (b) Falling rate. Asterisk (*) indicates a difference that is considered statistically significant compared with baseline values (Wilcoxon signed-rank test: *P* <0.05) and vertical bars represent the standard errors of means.

**Fig 8:**
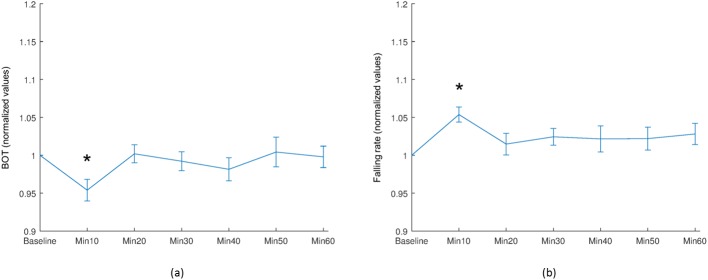
Selected mean waveform parameters of the tissue ONH. Selected mean waveform parameters of the tissue ONH. (a) BOT, and (b) Falling rate. Asterisk (*) indicates a statistically significant difference compared with baseline values (Wilcoxon signed-rank test: *P* <0.05) and vertical bars represent the standard errors of means.

A significant difference in the extent of skew was observed within the vascular region of the ONH at 40 minutes after water intake (*P* = 0.039), with a significant increase in falling rate at 50 minutes after water intake (*P* = 0.041), as shown in Fig 7. Furthermore, a significant decrease in BOT, and a significant increase in falling rate was observed in the tissue region of the ONH at 10 minutes after water intake (*P* = 0.011 and *P* = 0.001, respectively), as shown in Fig 8.

**Fig 9:**
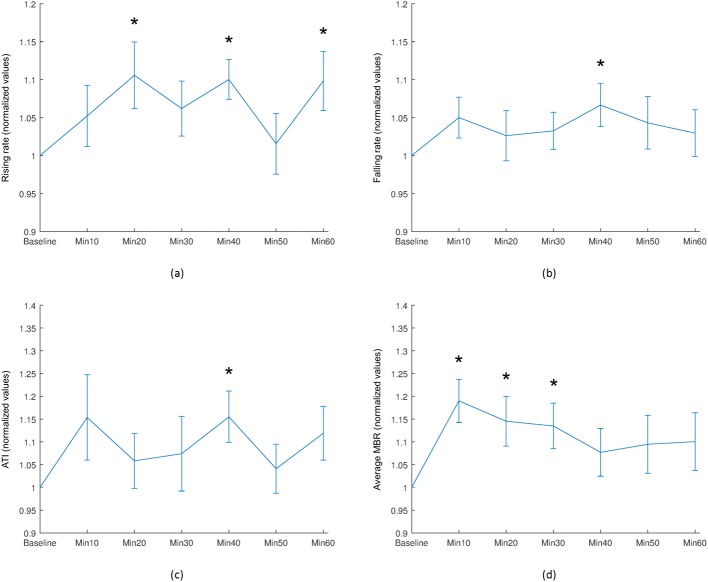
Selected mean waveform parameters of the avascular region away from the ONH. (a) Rising rate, (b) Falling rate, (c) ATI, and (d) Average MBR. Asterisk (*) indicates a statistically significant difference compared with baseline values (Wilcoxon signed-rank test: *P* < 0.05) and vertical bars represent the standard errors of means.

Significant increases in rising rate were also observed in the avascular region away from the ONH at 20 minutes (*P* = 0.026), 40 minutes (*P* = 0.003), and 60 minutes (*P* = 0.026) after water intake, as shown in Fig 9. A significant increase in both falling rate and ATI was observed at 40 minutes after water intake (*P* = 0.044, *P* = 0.039, respectively). A significant increase in MBR, indicating an increase in blood flow was observed at 10 minutes (*P* = 0.004), 20 minutes (*P* = 0.034), and 30 minutes (*P* = 0.020) after water intake.

The significant increase in the average MBR of the avascular region, observed 10–30 minutes after water intake, demonstrates ineffective autoregulation during this period. This suggests that autoregulation is active in the entire ONH, but may be less effective in areas that receive blood supply from the microcirculation and are located away from the ONH. In our study, significant increases in rising rate, falling rate, and ATI were all observed in the avascular region away from the ONH at 40 minutes after water intake. Increase in ATI and rising rate both reflect a reduction in the time required to reach the maximum blood flow value in a heartbeat, which corresponds with the period in which the eye was working under increased IOP due to water loading. Furthermore, rising rate has also been found significant at 20 and 60 minutes after WDT. Luft et al. have presented that the rising rate must be carefully considered pertaining to its moderate level of repeatability [50]. We have also observed an irregular trend for rising rate. Furthermore, the increase in falling rate and decrease in BOT observed in the tissue area of the ONH possibly reflect ocular autoregulatory mechanisms that become active following an increase in perturbation of IOP [48]. Previous studies have demonstrated the occurrence of choroidal autoregulation after an increase in IOP, including changes in arterial blood flow and perfusion pressure [54,55]. Our results revealed significant changes in blood flow up to 30 minutes after WDT in the choroidal microcirculation.

## Discussion

Many studies have used WDT as an explorative method for understanding ocular physiology in the context of both healthy and diseased conditions. However, the complete underlying mechanism of the effects of water intake on ocular physiology remains undetermined [45]. In this study, we investigated the hemodynamic responses of healthy human eyes during WDT. We assessed the pulse waveform parameters of hemodynamic response at the ONH to determine whether these could enable us to understand ocular physiology to predict risk of damage to ocular tissues.

As our study only included young healthy subjects, we expected effective autoregulation and no significant change in the blood flow. Our results confirmed no significant changes in mean MBR over the entire ONH, in the vasculature of the ONH and in the tissue area of the ONH, indicating that consistent blood flow was maintained throughout the WDT process. We observed a significant decrease in heart rate, as measured using the MBR waveform at all time points after drinking 1 liter of water, relative to baseline heart rate. This finding agrees with those of previous studies, in which a significant increase in blood pressure and a decrease in heart rate was reported in older study participants only (those 57 ± 2.2 years of age) [46], after intake of 1 liter of water. An increase in resting mean arterial blood pressure, with a decrease in resting heart rate was also reported after drinking 500 ml of water [47].

Reports from previous studies indicate that significant changes in IOP were observed during the first 15 minutes after water intake in healthy participants, and that this IOP remained elevated above baseline levels for 45 minutes after water intake [48]. In our study, we observed a significant increase in falling rate in the entire ONH, as well as in the tissue area of the ONH, based on data from LSFG recordings made at 10 minutes after water intake. However, no significant changes in falling rate were observed in the ONH vasculature. These findings indicate that falling rate in the entire ONH is based on changes in the tissue area subjected to WDT. A significant decrease in BOT was observed in the tissue area of the ONH at 10 minutes after water intake. Conversely, no significant changes in BOT were observed over the entire ONH. These observations may reflect an inability to maintain an elevated rate of blood flow in a tissue area. Significant increases in skew and falling rate were observed in the ONH vasculature at 40 and 50 minutes after water intake, respectively, although no significant changes in either of these parameters were observed in the tissue area or in the entire ONH. Therefore, when considering the results from the ONH region, we suggest that changes in IOP immediately after water intake might best be reflected in blood flow waveform parameters of the tissue region of ONH and whole ONH. Whereas, in the later stages, these changes may be better observed in the ONH vasculature only.

Tsuda et al. have shown that falling rate, skew, and BOT are significantly correlated with age in the Japanese population, both for tissue area and whole ONH region. [49]. Furthermore, they suggested that tissue area falling rate is the only independent factor indicating age. Luft et al. reproduced the same results for white subjects of Western European descent [50]. Our study also indicates significant increase in falling rate, and decrease in BOT in tissue area 10 minutes after water intake. Luft et al. have also suggested that the decrease in Windkessel effect with age may be reflected by falling rate and BOT. There may be a possibility that the significant change in the two parameters in our study is due to Windkessel effect. BOT is negatively correlated with systemic vascular resistance when measured invasively using Swan-Ganz catheter [51]. The decrease in tissue area BOT 10 minutes after water intake may reflect an underlying increase in systemic vascular resistance. Mechanisms that increase vasoconstriction and changes in viscosity of blood are responsible for causing increase in systemic vascular resistance. Cardio-ankle vascular index (CAVI) is yet another independent factor that contributes to skew and BOT [52,53]. Evaluation of CAVI in conjunction with pulse waveform parameters may also provide further information for patients suffering from cardiovascular disease.

Further research is required to compare results of MBR obtained after WDT in patients with glaucoma compared with those from participants without ocular pathologies. MBR when conducting LSFG recordings from the eyes of patients with glaucoma has been reported to be lower than that of the eyes of those without any ocular pathology. Furthermore, a statistically significant correlation between this parameter and thickness of the circumpapillary retinal nerve fiber layer has been reported, which may serve as an indicator of glaucoma [56]. As opposed to healthy subjects, normal tension glaucoma patients have significantly lower skew values and higher ATI [57]. Using WDT, ocular blood flow parameters may contribute to categorize normal tension glaucoma patients. The findings from this work can help researchers suggest the most effective waveform parameters in distinguishing between non-glaucomatous and glaucomatous eyes. Furthermore, monitoring of the changes in these parameters over time might also help in evaluating the progression of glaucoma.

The autoregulatory capacity of human eyes has been reported to decrease with advancing age [58]. The effects of aging on blood flow and the pulse waveforms of the ONH and avascular choroidal region after WDT should, therefore, be investigated in elderly individuals (aged ≥ 65 years old). A better understanding of the effects of aging may enable researchers to assess the autoregulatory capacity of eyes and the associated pathology of diseases. Further studies of changes in blood flow pulse waveform parameters in association with changes in IOP after WDT may also broaden our knowledge of the underlying heterogeneity of waveform characteristics between different individuals and demonstrate whether or not any correlation exists between these changes. Specifically, ocular waveform parameters could potentially be established as useful predictive biomarkers to assess the changes that take place in the ocular tissues of glaucomatous eyes using provocative tests such as WDT, and potentially help in researching for glaucoma treatments. These parameters may contribute to our understanding of the etiology of ocular conditions in longitudinal studies that aim to clarify changes in ocular tissue due to perturbation in perfusion pressure and the resulting changes in ocular blood flow.

To the best of our knowledge, this is the first study to investigate changes in ocular blood flow parameters during WDT. Further studies to investigate changes in ocular blood flow during WDT may also be conducted to correlate fluctuations in IOP with choroidal measures and ocular pathologies such as glaucoma.
